# Association of sweetened carbonated beverage consumption during pregnancy and ADHD symptoms in the offspring: a study from the Norwegian Mother, Father and Child Cohort Study (MoBa)

**DOI:** 10.1007/s00394-022-02798-y

**Published:** 2022-01-23

**Authors:** Liv Grimstvedt Kvalvik, Kari Klungsøyr, Jannicke Igland, Ida Henriette Caspersen, Anne Lise Brantsæter, Berit Skretting Solberg, Catharina Hartman, Lizanne Johanna Stephanie Schweren, Henrik Larsson, Lin Li, Ingeborg Forthun, Stefan Johansson, Alejandro Arias Vasquez, Jan Haavik

**Affiliations:** 1 Department of Global Public Health and Primary Care, University of Bergen, Bergen, Norway; 2 Department of Biomedicine, University of Bergen, Bergen, Norway; 3 Department of Clinical Science, University of Bergen, Bergen, Norway; 4 Department of Medical Genetics, Haukeland University Hospital, Bergen, Norway; 5 Division of Psychiatry, Bergen Center for Brain Plasticity, Haukeland University Hospital, Bergen, Norway; 6 Division of Mental and Physical Health, Norwegian Institute of Public Health, Oslo, Norway; 7 Centre for Fertility and Health, Norwegian Institute of Public Health, Oslo, Norway; 8 Department of Environmental Health, Norwegian Institute of Public Health, Oslo, Norway; 9 Interdisciplinary Center Psychiatry and Emotion Regulation (ICPE), University Medical Center Groningen, University of Groningen, Groningen, The Netherlands; 10 Child- and Adolescent Psychiatric Outpatient Unit, Hospital Betanien, Bergen, Norway; 11 School of Medical Sciences, Örebro University, Örebro, Sweden; 12 Department of Medical Epidemiology and Biostatistics, Karolinska Institutet, Stockholm, Sweden; 13 Department of Psychiatry, Donders Institute for Brain, Cognition and Behaviour, Radboud University Medical Center, Geert Grooteplein Zuid 10, 6525 GA Nijmegen, The Netherlands; 14 Department of Human Genetics, Donders Institute for Brain, Cognition and Behaviour, Radboud University Medical Center, Geert Grooteplein Zuid 10, 6525 GA Nijmegen, The Netherlands

**Keywords:** ADHD, Maternal nutrition, MBRN, MoBa, Neurodevelopmental disease, Pregnancy, Sweetened carbonated beverages

## Abstract

**Purpose:**

Intrauterine exposures influence offspring health and development. Here we investigated maternal intake of sweetened carbonated beverages (SCB) during pregnancy and its association with ADHD symptoms in the offspring.

**Methods:**

This study was based on the Norwegian Mother, Father and Child Cohort Study (MoBa) and the Medical Birth Registry of Norway. Maternal diet mid-pregnancy was assessed using a food frequency questionnaire (FFQ). All mothers who responded to the FFQ and a questionnaire when their child was 8 years of age were included (*n* = 39,870). The exposure was defined as maternal intake (daily servings) of SCB, using no daily intake as reference. Outcome was offspring ADHD symptoms, evaluated as a continuous standardized ADHD score and as a binary outcome of six or more ADHD symptoms vs. five symptoms or less. Associations were analysed using log-binomial regression and linear mixed regression models with adjustment for covariates.

**Results:**

The adjusted regression coefficients for the standardized ADHD offspring symptom score were 0.31 [95% confidence intervals (0.001, 0.62)] and 0.46 (0.15, 0.77) for maternal daily intake of ≥ 1 glasses of SCB, when the models included adjustments for total energy intake or energy intake from other sources than SCBs and sweet drinks, respectively. The corresponding adjusted relative risks were 1.16 (1.004, 1.34) and 1.21. (1.05, 1.39) for drinking ≥ 1 glasses daily.

**Conclusion:**

In a large pregnancy cohort with offspring followed until 8 years of age, we found an association between maternal daily intake of SCB and offspring ADHD symptoms. These results suggest a weak positive relationship between prenatal exposure to SCB and offspring ADHD.

**Supplementary Information:**

The online version contains supplementary material available at 10.1007/s00394-022-02798-y.

## Introduction

Neurodevelopmental disorders typically become apparent during childhood but are also an important cause of long-term disability [1]. Attention-deficit/hyperactivity disorder (ADHD) is the most common of these conditions, with prevalence estimates around 3–4% among children and adolescents in Norway [1–3] and worldwide prevalence around 5% in children and 2.5% in adults [4]. ADHD is defined according to a set of characteristic symptoms, including hyperactivity, inattention and impulsivity [5, 6]. These are dimensional traits, observed at various levels in all humans. To qualify for an ADHD diagnosis, these traits, or symptoms, must be age inappropriate and extreme in such a way that they interfere with and impair a person’s daily life; at school, at work or in social settings.

ADHD is a complex multifactorial disorder where heritability is estimated to be approximately 74% based on twin studies [7]. The heritability of less than 100% indicates that both genes and environment contribute to its development. A recent genome-wide association study of ADHD identified 12 significant loci, and taking all common variants into account, approximately 22% of the heritability was explained [8]. In terms of neurobiological background, some findings point at alterations of the dopaminergic system as being associated with ADHD [9]. Reduced striatal and hippocampus volumes haves been found among patients with ADHD, with most pronounced findings in childhood, suggesting brain maturation delay as a component in ADHD [10, 11]. Several perinatal risk factors are associated with later ADHD [12]. The identification of modifiable early-life risk factors, including nutritional factors, is also important, as such information could potentially offer opportunities for primary prevention. So far, such possibilities have been less studied in relation to ADHD. A recent study from the Norwegian Mother and Child Cohort Study, however, found that overall maternal diet quality and lower intake of ultra-processed foods were weakly associated with the child’s ADHD symptoms at 8 years of age [13].

The high dietary intake of sugar in food and beverages in Western diets has raised much concern. Soft drinks (soda or fizzy drinks) are non-alcoholic carbonated beverages either sugar-sweetened or artificially sweetened. In Norway, sales of carbonated beverages have increased tenfold since the 1950s. The sales of sugar-sweetened soft drinks increased until the millennium after which it decreased, while sales of artificially sweetened carbonated beverages have gradually increased [14]. High maternal intake of sucrose and sugar-sweetened beverages during pregnancy has been linked to adverse outcomes in human offspring, as well as in animal models. In pregnant mice, prenatal sucrose diluted water has been associated with changes in the dopaminergic system and increased impulsivity and decreased attention [15]. In humans, a relatively small study reported that maternal sucrose consumption was negatively associated with childhood cognition scores at 7 years of age [16]. Sugar-sweetened beverages during pregnancy have also been associated with other outcomes, such as preterm delivery [17] and congenital heart defects [18].

Based on data from a large cohort study, we aimed to investigate whether intake of sugar- or artificially sweetened carbonated beverages (SCB) during pregnancy is associated with increased ADHD symptoms in offspring at 8 years of age. Previous studies have not been able to account for unobserved confounding factors that could affect the association between maternal intake of SCBs and hyperactivity in children. By using a sibling comparison design, thus controlling for shared characteristics that remain stable between pregnancies, it is possible to better assess the role of such confounding factors.

### Materials and methods

### Study design and study sample

This study was based on data from the Norwegian Mother, Father and Child Cohort Study (MoBa) and the Medical Birth Registry of Norway (MBRN). MoBa is a large prospective population-based pregnancy cohort study conducted by the Norwegian Institute of Public Health. Participants were recruited from across Norway during 1999–2008 [19]. The women consented to participation in 41% of the invited pregnancies. Participating women answered three questionnaires during pregnancy, of which the second (Q2) was a food frequency questionnaire (FFQ), introduced in 2002, and completed during mid-pregnancy. After delivery, questionnaires were forwarded to the family when the child was 6 months, 18 months, and at 3, 5, 7 and 8 years, as well as further questionnaires when the child is a teenager. The cohort now includes 114,500 children, 95,200 mothers and 75,200 fathers. For the MoBa pregnancies, data are routinely linked to the MBRN, a national health registry based on mandatory reporting of information on pregnancies and birth outcomes for all births in Norway since 1967 [20]. The establishment of MoBa and initial data collection was based on a license from the Norwegian Data protection agency and approval from The Regional Committee for Medical Research Ethics. The MoBa cohort is currently regulated by the Norwegian Health Registry Act. The present study was approved by The Regional Committee for Medical Research Ethics (2015/2055) and is based on version 12 of the quality-assured data files released for research in January 2019.

**Fig. 1:**
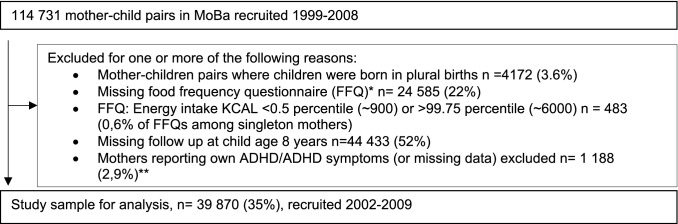
Flowchart of the study population. *The MoBa food frequency questionnaire was introduced in March 2002. **1013 mother–child pairs were mother report having ADHD or ADHD symptoms were included in a secondary analysis

Mother–child pairs in singleton births, who had answered the FFQ during pregnancy as well as completed a questionnaire when the child was 8 years of age (Q8) were included in our study sample. Exclusions from the total study population of 114,731 mother–child pairs were made for one or more of the following reasons: children born in plural births (4%), missing FFQ (22%), calculated daily energy intake < 0.5 percentile (~ 900 kcal) or > 99.75 percentile (~ 6000 kcal) (0.6% of FFQs among singleton mothers), missing follow-up when child was 8 years (52%). In the main analyses we also excluded mothers who reported own ADHD/ADHD symptoms (3%), to exclude obvious genetic contribution to their child’s ADHD symptoms. Women report their own ADHD/ADHD symptoms in questionnaire 6 (Q6, when the child is 3 years old) by the Adult ADHD Self-Report Scale (ASRS) and in Q8 (when the child is 8 years of age) by answering either having had or having the ADHD diagnosis. In the main analyses, we excluded women reporting ever having had an ADHD diagnosis and/or having a score ≥ 14 on the ASRS, as well as women missing information on maternal ADHD (from both Q8 and Q6). In secondary analyses, these women were included, and maternal ADHD was adjusted for in the statistical model. These exclusions resulted in a final study population of 35,309 unique mothers contributing 39,987 mother–child pairs for the main analyses (Fig. 1). There were 4414 mothers that participated with more than one pregnancy, yielding a total of 8975 siblings. For the analyses where women with own ADHD were included, the study population was 40,883 mother–child pairs.

### Exposure assessment and definition

In the period between 2002 and 2008, maternal diet was assessed in MoBa through a FFQ covering habitual diet during the first half of pregnancy [21]. Respondents were instructed to report their average intake since they became pregnant. There were 39 questions about beverages, of which eight were considered relevant for the current study. These included five questions about intake of SCB (i.e., sugar-sweetened cola drinks, other sugar-sweetened soft drinks, energy drinks and artificially sweetened cola and other soft drinks), two questions about non-carbonated sweet drinks (i.e., sugar-sweetened and artificially sweetened cordial/fruit syrup), and one question about nectar. The answer options were given as servings (cups or glasses) per day (between 1 and 8), servings per week (between 1–2 and 5–6), or servings per month (between 0 and 2–3). A serving was defined as 200 mL for nectar and 250 mL for the remaining beverages [22]. In Norway, orange juice is considered 100% liquid fruit, while nectar is fruit juice with added sugar and water. Orange juice was therefore not included among sugar-sweetened drinks in this study. The main exposure variable for the current study was intake of SCB (sugar- and artificially sweetened) combined and categorized into less than one daily serving (reference group) and 1 serving or more daily. As secondary analyses, we separated sugar-sweetened SCB and artificially sweetened SCB. In supplementary analyses we additionally categorized the main exposure variable of SCB intake as less than one daily serving, 1 serving daily and 2 servings or more daily. Further to evaluate the intake of sweet cold drinks overall, SCBs, nectars and fruit syrup were combined into one exposure variable in supplementary analyses.

### Definition of the outcome

Questionnaire 8 (Q8) focused on the child’s behaviour and was mailed to the mothers when the child was approximately 8 years of age. Child ADHD symptoms were assessed by 18 questions from the Parent/Teacher Rating Scale for Disruptive Behaviour Disorders (RS-DBD) addressing ADHD symptoms [23]. ADHD symptoms are rated by the mother on a four-point Likert scale (1 = never/rarely, 2 = sometimes, 3 = often, 4 = very often). We defined the outcome “ADHD symptoms in offspring” in two ways: first, we calculated the average score for the mentioned 18 questions for each child and scaled the responses to a *T* score with a mean of 50 and standard deviation of 10, similar to a previous study from MoBa by Torvik et al. [24]. We required 9 or more answers to the 18 questions to get valid outcome values. Second, scores were also dichotomized using six or more symptoms of inattentiveness and/or hyperactivity occurring often or very often as cut-off, in accordance with the DSM criteria (six or more of nine symptoms/traits). For the dichotomized version of the outcome, we required 5 or more answers to the 9 questions about both inattentiveness and hyperactivity to get valid outcome values.

### Other variables

Maternal educational level, maternal pre-pregnancy body mass index (BMI), maternal depression and anxiety, components of the maternal diet (obtained from MoBa) and maternal age at delivery, parity, birth year and season (obtained from the MBRN) were a priori considered as possible confounders for the association between maternal intake of SCB and offspring ADHD symptoms. Maternal educational level was categorised into low (less than high school), middle (high school), or high (4 years or more of college/university). Pre-pregnancy BMI was categorized into < 18.5 (underweight), 18.5–24.9 (normal weight), 25–29.9 (overweight) and 30 kg/m^2^ or more (obesity). Maternal depression and anxiety symptoms were assessed around gestational week 30 (Questionnaire 3) using selected items from the Hopkins Symptoms Checklist-25 where four questions concern symptoms of anxiety and four concern symptoms of depression. Response categories are "not bothered" "a little bothered" "quite bothered" and "very bothered" and we used the mean score as a continuous adjustment variable. Maternal age was categorised into < 20, 20–24, 25–29, 30–34 and 35 years or older. Parity was categorized into nulliparous, 1 and 2 or more previous pregnancies. Birth season was categorized into January–March, April–June, July–September, October–December. Offspring sex (obtained from MBRN) was used for stratified analyses.

Other components of the maternal diet were added as adjustment variables. Calculated total maternal fiber intake (continuous variable) served as a proxy for a healthy diet [25]. We also adjusted for total energy intake (as a continuous variable) in addition to maternal intake of non-carbonated sweet drinks (number of servings of nectars and fruit syrup daily categorized into “less than 1” and “1 or more daily servings”). Maternal intake of alcohol may be associated with both SCB and offspring ADHD. Maternal intake of alcohol during pregnancy was therefore evaluated as a potential confounder and adjusted for in secondary analyses, categorized into less than monthly intake and monthly or more.

Household behaviour relating to SCBs could be a possible confounder for the relation between intake during pregnancy and child ADHD symptoms. The child’s intake of sweet beverages in early childhood could be viewed as a proxy for household behaviour. The questionnaire at child’s age 3 years (Q6) included questions about the child’s diet, of which two asked about frequency (but not volume) of intake of sugar-sweetened and artificially sweetened beverages. These questions did not distinguish between carbonated and non-carbonated beverages (sweet drinks other than fruit juice, nectar, carbonated drinks). The child’s intake of sugar-sweetened beverages and artificially sweetened beverages was categorized into two separate variables (“less than daily intake”, “1 time daily” and “2 or more times daily”) and included as adjustment variables in secondary analyses. Mother–child units that either missed the whole questionnaire (*n* = 5739) or the specific variable on child’s intake of beverages in Q6 were excluded from this analysis.

### Statistical analysis

The association between maternal intake of SCB during pregnancy and the standardized ADHD scores as outcome was estimated using a linear mixed regression model, intake of SCB as fixed-effect and random intercept for mother to account for repeated pregnancies to the same mother. Robust standard errors were used to account for heteroskedasticity.

For the dichotomous outcome, a general linear model with a log-link and binomial distribution was used to estimate relative risks (RR) with 95% confidence intervals (CI). Clustered robust standard errors were used to account for women participating with multiple pregnancies. The reference category was non-drinkers of SCB (not drinking any carbonated sugar-sweetened or artificially sweetened beverages). Due to convergence problems with the log-binomial regression model, we used a modified Poisson regression approach with a robust error variance procedure [26] to estimate RR when adjustment for maternal depression and anxiety scores were included in the model (Supplementary table 1).

Crude and adjusted models with different sets of adjustment variables are presented in the tables to illustrate how various covariates influenced the estimates. Adjustments for total energy intake was evaluated in a separate model. SCBs are part of the total composition of the diet. Models without adjustments for total energy intake estimate the total effect of our exposure (maternal intake of SCB) on the outcome (offspring ADHD symptoms), while adjusting for total energy intake confers a situation where the exposures were substituted by other nutritional components but still result in the same total energy intake [27]. Because it has been reported that sugar-sweetened beverages does not contribute to the same satiation as non-liquid calories and result in higher total energy intake [28, 29], we also conducted additional analyses where we adjusted for energy coming from other sources than SCB, nectars and fruit syrup.

There is a loss to follow-up from pregnancy (Q2) to the child is 8 years of age (Q8) in MoBa. We therefore used inverse probably weighting, previously suggested as a method to account for attrition bias [30, 31], to evaluate whether the loss to follow-up had potential to affect the associations studied. We calculated participation weights for the follow-up at 8 years based on simple participation probabilities; the number of mothers in a population subgroup in the study sample divided by the number of mothers in the same subgroup participating at Q2. The weights were created based on maternal age, parity and educational level, which are factors related to participation and loss to follow-up in MoBa [31].

Several women participated in MoBa twice or more, which made it possible to correct for unmeasured confounding shared by siblings. We analysed the association among siblings who were discordant on the exposure (i.e. having different values for maternal intake of SCB during pregnancy, *n* = 913 siblings), using a sibling comparison design with a fixed-effect model (xtreg command with fe option in Stata). We included all maternal siblings among mothers participating with at least two pregnancies, identified by mothers’ study identification number (plural births were already excluded from the study population). For the sibling analyses, we examined the associations with the outcome as a continuous variable (standardized), and present estimates adjusted for covariates.

### Secondary analyses

We conducted a series of secondary analyses to identify possible explanatory factors and vulnerable subpopulations.

Offspring intake of sweet beverages in early childhood could represent both a confounder as a proxy for household behaviour, but also as an intermediate factor between maternal SCB intake and offspring ADHD symptoms. Our main aim of this study was to investigate the potential total effect of maternal intake of SCB on offspring ADHD symptoms; without separating/disentangling direct and indirect effects. We therefore chose not to adjust for intermediate factors (i.e. the child’s intake of sweetened beverages) in the main analyses, but we report this as a secondary analysis. The relationship between the described factors is presented graphically in a directed acyclic graph (DAG) in Supplementary Fig. 1.

We evaluated maternal alcohol intake in pregnancy as a potential confounder and found that women drinking alcohol “monthly or more often” had a relative risk of 0.84 (0.77–0.92) for drinking “1 serving or more daily” of SCB and a regression coefficient of 0.99 (0.68–1.30) for offspring ADHD symptoms at 8 years of age. In secondary analyses we therefore adjusted for maternal intake of alcohol.

To evaluate whether the association with offspring ADHD symptoms differed between maternal intake of sugar-sweetened and artificially sweetened carbonated beverages, we divided SCB into sugar-sweetened and artificially sweetened carbonated beverages, and analysed their association with offspring ADHD symptoms, using no intake of the specific beverage as reference group, and additionally adjusting for the other beverage.

In a further secondary analysis, we examined a possible association to sweet not-carbonated drinks. While other sweet beverages, such as nectars and fruit syrup, were one covariate in our adjusted models, we also included a model were SCBs were combined with these drinks in a combined exposure variable.

In a final secondary analysis, we included 1013 women who reported having past or present an ADHD diagnoses or ADHD symptoms. In the models, we adjusted maternal ADHD as a confounding factor.

The main analyses were conducted in STATA version 16.1 (College Station, TX), while the weights created for the inverse probability weighting were made using RStudio version 1.4.1717 and R version 3.4.4 [32].

## Results

### Study population

In our study sample of mother–child, units followed up until offspring age 8 years (*n* = 39,970), 87% (*n* = 34,571) of the mothers reported no daily intake of SCB during pregnancy, while 13% (*n* = 5299) reported 1 serving or more of SCB (Table 1).

**Table 1:** Daily intake of sweetened carbonated beverages by participant characteristics

	Study population (in Q2 and Q8)	Sweetened carbonated beverages during pregnancy (daily intake)		
		< 1 daily serving		Daily (1 or more servings)
	*n* (column %)	*n* (row %)		
Mothers-child units	39,870 (100)	34,571 (86.7)	5299 (13.3)	
Maternal age at delivery (years)^a^		*n* (column %)		
< 20	176 (0.4)	125 (0.4)	51(1.0)	
20–24	2925 (7.3)	2418 (7.0)	507 (9.6)	
25–29	12,801 (32.1)	11,136 (32.2)	1665 (31.4)	
30–34	16,344 (41.0)	14,231 (41.2)	2113 (39.9)	
35 years and older	7624 (19.1)	6661 (19.3)	963 (18.2)	
Maternal parity^a^				
0	18,273 (45.8)	16,148 (46.7)	2125 (40.1)	
1	14,064 (35.3)	12,021 (34.8)	2043 (38.6)	
2+	7533 (18.9)	6402 (18.5)	1131 (21.3)	
Maternal education^b^				
Less than high school	599 (1.5)	456 (1.3)	143 (2.7)	
High school	10,903 (27.4)	8868 (25.7)	2035 (38.4)	
4 years or more of college/university	27,522 (69.0)	24,519 (70.9)	3003 (56.7)	
Missing education information	846 (2.1)	728 (2.1)	118 (2.2)	
Pre-pregnancy body mass index (BMI)^b^				
< 18.5	1060 (2.7)	921 (2.7)	139 (2.6)	
18.5–24.9	26,158 (65.6)	23,397 (67.7)	2761 (52.1)	
25–29.9	8470 (21.2)	7031 (20.3)	1439 (27.2)	
30 or higher	3232 (8.1)	2417 (6.7)	815 (15.4)	
Missing BMI information	950 (2.4)	805 (2.3)	145 (2.7)	
Alcohol intake less than monthly	35,377 (88.7)	30,591 (88.5)	4786 (90.3)	
Alcohol intake monthly or more often	4493 (11.3)	3980 (11.5)	513 (9.7)	
Maternal depression and maternal anxiety symptoms [mean score (SD)]^c^	1.2 (0.3)	1.2 (0.3)	1.3 (0.4)	

^a^Obtained from the Medical Birth Registry of Norway

^b^Obtained from the baseline questionnaire Q1 in MoBa

^c^*n* = 824 missing in these analyses

The majority (60.1%) of mothers were older than 30 years when they gave birth, 45.8% were nulliparous and 69.0% had higher education (4 years or more of college/university). Mothers with one or more daily servings of SCB where younger, had lower education, higher BMI and a lower proportion of nulliparous mothers compared to mothers with less than one daily serving. Mothers with one or more daily servings of SCB also slightly less often drank alcohol monthly or more often than mothers with less than one daily serving of SCB (Table 1).

### Primary analyses

For the standardized ADHD score, the regression coefficient for offspring of mothers who drank 1 or more serving of SCB daily in pregnancy compared to mothers with no daily intake of SCB was 0.87 (95% confidence intervals (CI) 0.57–1.17). Adjusting for covariates attenuated the regression coefficients, and further attenuation was observed when also adjusting for total energy intake (Table 2). However, there was still an increase in standardized ADHD scores in offspring to mothers who drank 1 or more servings of SCB daily compared to none (0.31; 0.001, 0.62). When adjusting for energy coming from other sources than sweet drinks (SCBs, nectar and fruit syrup), the estimate was slightly higher (0.46; 0.15, 0.77) for offspring to mothers drinking 1 or more servings daily. When splitting daily servings of SCBs into 1 serving daily and 2 or more servings daily, there was no association between 1 serving daily (− 0.01; − 0.48, 0.46) and offspring ADHD symptoms in the fully adjusted model, while the coefficient was 0.49 (0.10, 0.87) for women drinking 2 servings or more daily (Supplementary table 2).

**Table 2:** Regression coefficients and 95% CIs from linear mixed models for the association between maternal intake of sweetened carbonated beverages (SCB) and a standardized ADHD offspring symptoms score

SCB (daily intake)	Mean score (SD)	Unadjusted regression coefficient (95% CI)^a^	Adjusted coefficient (95% CI)^b^	Adjusted coefficient (95% CI)^c^	Adjusted coefficient (95% CI)^d^	Sibling comparison adjusted coefficient (95% CI)^e^	Sibling comparison adjusted coefficient (95% CI)^f^
< 1 serving daily	49.61 (9.53)	0 (ref)	0 (ref)	0 (ref)	0 (ref)	0 (ref)	0 (ref)
1 serving or more daily	50.51 (10.40)	0.87 (0.57, 1.17)	0.56 (0.25, 0.87)	0.31 (0.001, 0.62)	0.46 (0.15–0.77)	0.69 (− 0.36, 1.74)	0.66 (− 0.38, 1.71)

Total *n* = 39,760 (for mother–offspring units to join the analyses 9 or more out of 18 questions on ADHD symptoms were required to be answered. For *n* = 110 (0.3%), the mothers had answered eight or less questions and these mother–offspring units were excluded from the analyses)

^a^Unadjusted regression coefficient from linear mixed model with the ADHD scores standardized to a mean of 50 and a standard deviation of 10 as outcome, intake of SCB as fixed-effect and random intercept for mother (*n* = 39,760)

^b^Regression coefficient from linear mixed model adjusted for daily intake of other sweet beverages such as nectars and fruit syrup, total fiber intake, maternal education, age, parity, prepregnancy BMI, maternal depression and anxiety at gestational week 30, birth year and birth season (*n* = 37,353 complete cases)

^c^As above but additional adjustment for total energy intake (kcal)

^d^As above but additional adjustment for total energy intake other than contributed from SCBs, nectars and fruit syrup

^e^Sibling comparison model adjusting for daily intake of other sweet beverages such as nectars and fruit syrup, total fiber intake, maternal education, age, parity, prepregnancy BMI, birth year and birth season, total energy intake (kcal) and maternal depression and anxiety at gestational week 30

^f^Sibling comparison model adjusting as above in (e) but for energy intake other than SCBs and nectars (kcal) instead of total energy adjustment

In women who reported no daily intake of SCB, the absolute risk of the child having six or more ADHD symptoms at 8 years of age was 3.4%, while the absolute risks in women drinking 1 or more servings daily of SCB was 4.5% (Table 3). The corresponding unadjusted RRs for offspring having six or more ADHD symptoms were 1.32 (1.15, 1.52). When adjusting for daily intake of other sweet beverages (not carbonated), total fiber intake, maternal education, age at delivery, parity, pre-pregnancy BMI, birth year and birth season, the RR was slightly attenuated (RR 1.24; 1.07–1.43). When additionally adjusting for total energy intake or adjusting for energy intake from other sources than sweet drinks, the RRs were RR 1.16 (1.004, 1.34) and 1.21 (1.05, 1.39), respectively.

**Table 3:** Relative Risk of offspring ADHD symptoms (6 or more) at 8 years of age by maternal daily intake of sweetened carbonated beverages (SCB) during pregnancy

Maternal intake of SCB beverages during pregnancy (daily intake)	Total (mother–child pairs)	*N* (%) offspring with 6 or more ADHD symptoms	Relative risk (RR) of offspring having 6 or more ADHD symptoms at 8 years of age (95% CI)					
			Unadjusted RR (95% CI)	Adjusted RR (95% CI)^a^	Adjusted RR (95% CI)^b^	Adjusted RR (95% CI)^c^	Inverse probability weighting adjusted RR (95% CI)^d^	Inverse probability weighting adjusted RR (95% CI)^e^
< 1 serving daily	34,469	1165 (3.4)	1 (reference)	1 (reference)	1 (reference)	1 (reference)	1 (reference)	1 (reference)
1 or more servings daily	5277	236 (4.5)	1.32 (1.15, 1.52)	1.24 (1.07, 1.43)	1.16 (1.004, 1.34)	1.21 (1.05, 1.39)	1.15 (1.00, 1.34)	1.20 (1.04, 1.39)

Total *n* = 39,746. Offspring with less than six symptoms are the reference group 124 (0.3%) mother–child units were excluded from these analyses due to missing answers on ADHD symptoms. For the dichotomized version of the outcome we required five or more answers to nine questions about both inattentiveness and hyperactivity to get valid outcome values

^a^Adjusted for daily intake of other sweet beverages such as nectars and fruit syrup, total fiber intake, maternal education, age, parity, pre-pregnancy BMI, birth year and birth season and using the vce cluster command to take account of mother participating several times with different pregnancies

^b^As above but additional adjustment for total energy intake

^c^Adjusted as in model a but with additional adjustment for energy intake from other than SCBs, nectars and fruit syrup

^d^Inverse probability weighting adjusting as above in model b

^e^Inverse probability weighting adjusting as above in model c

When maternal depression and anxiety symptoms were included as additional confounders, similar RR estimates to those shown in the adjusted model in Table 3 were seen (Supplementary table 1).

There was a loss to follow-up for mothers answering the questionnaires from baseline (Q1) to Q8. The distribution of various characteristics for women with singleton pregnancies completing Q1, Q2 and Q8 is described in Supplementary Table 3. Mothers who completed both Q2 and Q8 were more often nulliparous, older and had high education compared to population who answered the baseline Q1. However, when we reanalysed the association between maternal intake of SCB and offspring ADHD symptoms using inverse probability weighting for loss to follow-up, results were very similar to the overall adjusted relative risk analyses (Table 3).

In the sibling comparison analysis, using a fixed-effect model to analyse offspring discordant for the exposure (maternal SCB intake during pregnancy), the adjusted regression coefficient for the association between SCB intake and offspring standardized ADHD scores were in the same direction and even larger than in the main analyses; however, given the low statistical power in this analysis, the estimate for women drinking 1 servings or more had a confidence interval that included zero (0.69; − 0.36, 1.74); see Table 2.

### Secondary analyses

Adjusting for offspring’s intake of sweetened beverages at 3 years of age, the beta coefficients for women drinking 1 serving or more was 0.08 (− 0.26, 0.42) when the full model was adjusted for total energy intake and 0.22 (− 0.12, 0.56) when the full model was adjusted for other sources of energy than SCBs, nectars and fruit syrup. The corresponding RR estimate for women with 1 or more serving SCB daily was 1.10 (0.93, 1.30) adjusted for the same variables as above (except for maternal depression and anxiety symptoms) and total maternal energy intake (data not tabulated).

We included maternal intake of alcohol during pregnancy as an adjustment variable in addition to intake of other sweet beverages, total fiber intake, maternal education, age at delivery, parity, pre-pregnancy BMI, birth year and birth season, total energy and maternal depression and anxiety, with hardly any change to the regression coefficients. After adjustment, the regression coefficient was 0.33 (0.02, 0.64) for 1 or more daily servings of SCB compared to less than one daily SCB serving. When adjusting for energy from other sources than SCBs, nectars or fruit syrup instead of total energy, the estimate was 0.47 (0.17, 0.78).

We also split maternal intake of SCB into sugar-sweetened and artificially sweetened carbonated beverages to compare their associations with offspring ADHD symptoms, using less than daily intake of the specific beverage as reference group, and additionally adjusting for the other beverage (Table 4). Among women drinking sugar-sweetened carbonated beverages daily, there was a higher proportion (11.0%) who also drank artificially sweetened carbonated beverages daily, than among women among women not drinking sugar-sweetened carbonated beverages daily (7.8%). In the fully adjusted models (including adjustment for total energy intake), the beta coefficient for ADHD symptoms was lower than in the combined SCB model (0.31; 0.001, 0.62) for women drinking 1 serving or more daily of sugar-sweetened carbonated beverages (0.15; − 0.33, 0.62), while it was similar for women drinking artificially sweetened beverages (0.30; − 0.08, 0.67). In the models without adjustment for total energy intake, maternal daily intake of 1 or more serving of sugar-sweetened SCB was associated with offspring ADHD symptoms (regression coefficient 0.68 (0.22, 1.14), while the regression coefficient for intake of artificially SCB was weaker (0.32; − 0.06, 0.69). When adjusting for energy intake from other sources than SCBs, nectar and fruit syrup instead of total energy intake, the coefficient was less attenuated for women drinking 1 or more sugar-sweetened SCB daily (0.51; 0.05, 0.97), while there was less change for women drinking 1 or more artificially sweetened SCB daily (0.28; − 0.1, 0.65). For total adjustment variables, see footnotes to the table.

**Table 4:** Regression coefficients and 95% CIs from linear mixed models for the association between maternal intake of sweetened carbonated beverages (SCB) and a standardized ADHD offspring symptoms score, showing results for total SCB and for sugar-sweetened SCB and artificially sweetened SCB

Daily SCB intake during pregnancy	Unadjusted regression for exposure coefficient (95% CI) (total SCB)^a^	Adjusted coefficient (95% CI) (total SCB)^b^	Adjusted coefficient (95% CI) sugar- sweetened SCB without total energy intake adjustment^c^	Adjusted coefficient (95% CI) sugar-sweetened SCB with total energy intake adjustment^d^	Adjusted coefficient (95% CI) sugar- sweetened SCB with adjustment for other energy intake than from SCBs, nectars and fruit syrup^e^	Adjusted coefficient (95% CI) artificially sweetened SCB without total energy intake adjustment^f^	Adjusted coefficient (95% CI) artificially sweetened SCB with total energy intake adjustment^g^	Adjusted coefficient (95% CI) artificially sweetened SCB with adjustment for other energy intake than from SCBs, nectars and fruit syrup^h^
< 1 serving daily	0 (ref)	0 (ref)	0 (ref)	0 (ref)	0 (ref)	0 (ref)	0 (ref)	
1 serving or more daily	0.87 (0.57, 1.17)	0.31 (0.001, 0.62)	0.68 (0.22, 1.14)	0.15 (-0.33, 0.62)	0.51 (0.05, 0.97)	0.32 (− 0.06, 0.69)	0.30 (− 0.08, 0.67)	0.28 (− 0.10, 0.65)

Total *n* = 39,760 (for mother–offspring units to join the analyses 9 or more out of 18 questions on ADHD symptoms had to be answered. For *n* = 110 (0.3%), the mothers had answered eight or less questions and these mother–offspring units were excluded from the analyses

^a^Unadjusted regression coefficient from linear mixed model with the ADHD scores standardized to a mean of 50 and a standard deviation of 10 as outcome, intake of SCB as fixed-effect and random intercept for mother to account for mothers participating in MoBa with more than one pregnancy

^b^Regression coefficient from a linear mixed model adjusted for daily intake of other sweet beverages such as nectars and fruit syrup, total fiber intake, maternal education, age, parity, prepregnancy BMI, maternal depression and anxiety at gestational week 30, birth year and birth season and total energy intake (kcal)

^c^Adjusted coefficient (95% CI) from a linear mixed model with the ADHD scores standardized to a mean of 50 and a standard deviation of 10 as outcome, intake of sugar-SCB as fixed-effect and random intercept for mother. Adjusted as above but additional adjustment for intake of artificially SCB and no adjustment for total energy intake (kcal)

^d^Adjusted coefficient (95% CI) from a linear mixed model with the ADHD scores standardized to a mean of 50 and a standard deviation of 10 as outcome, intake of sugar-SCB as fixed-effect and random intercept for mother. Adjusted as above but additional adjustment total energy intake (kcal)

^e^Adjusted coefficient (95% CI) from a linear mixed model with the ADHD scores standardized to a mean of 50 and a standard deviation of 10 as outcome, intake of sugar-SCB as fixed-effect and random intercept for mother. Adjusted as above but additional adjustment for other energy intake than from SCBs, nectars and fruit syrup

^f^Adjusted coefficient (95% CI) from a linear mixed model with the ADHD scores standardized to a mean of 50 and a standard deviation of 10 as outcome, intake of artificially SCB as fixed-effect and random intercept for mother. Adjusted as above but additional adjustment for intake of sugar-SCB and no adjustment for total energy intake (kcal)

^g^Adjusted coefficient (95% CI) from a linear mixed model with the ADHD scores standardized to a mean of 50 and a standard deviation of 10 as outcome, intake of artificially SCB as fixed-effect and random intercept for mother. Adjusted as above but additional adjustment for total energy intake (kcal)

^h^Adjusted coefficient (95% CI) from a linear mixed model with the ADHD scores standardized to a mean of 50 and a standard deviation of 10 as outcome, intake of artificially SCB as fixed-effect and random intercept for mother. Adjusted as above but additional adjustment for other energy intake than from SCBs, nectars and fruit syrup

In further secondary analyses, we included SCBs, nectars and fruit syrup as a combined exposure (Supplementary table 4). In a fully adjusted model (including total energy intake), there was no evident association between daily servings of 1 or more sweet beverage and offspring ADHD symptoms at 8 years of age (− 0.03; − 0.24, 0.17).

Finally, we included mothers with present or previous ADHD symptoms or diagnosis in the study population and adjusted for maternal ADHD. The fully adjusted (including total energy intake) regression coefficients for offspring ADHD symptoms at 8 years of age were 0.36 (0.05, 0.70) for women drinking 1 or more serving of SCB daily, compared to mothers who drank less than 1 serving SCB daily. The corresponding RR estimate for offspring having six or more symptoms at 8 years for mothers drinking 1 or more SCB daily was 1.19 (1.04, 1.36) (data not tabulated).

## Discussion

In this large population-based cohort study, we found that daily intake of 1 or more serving of SCB as compared to less than 1 serving daily during mid-pregnancy was associated with a weak increase in ADHD symptom score and an increased risk of offspring having six or more ADHD symptoms at 8 years of age. Given that the standardized ADHD score had a mean of 50 and a standard deviation of 10 in the total study population, estimated regression coefficients in the range of 0.3–0.8 for difference between exposed women in different adjustment models should be considered as weak effects. The adjusted relative risks of offspring having 6 or more ADHD symptoms were around 1.2, with crude absolute risks at 4.5 and 3.4% in offspring to mothers who drank 1 or more servings SCB daily and less than 1 serving daily, respectively. In comparison, preterm birth, which is an established perinatal risk factor for ADHD, has a reported odds ratio of offspring receiving ADHD medication of 2.1 (1.4, 2.7) for infants born between 23 and 28 gestational weeks and 1.6 (1.4, 1.7) for those born between 29 and 36 weeks, compared to infants born at 39–41 weeks' gestation [33]. Relative risks of having 6 or more ADHD symptoms in the current study are thus moderate compared to this perinatal exposure.

After taking demographic and socioeconomic factors into account, the association between maternal intake of 1 or more serving of SCB daily and a continuous, standardized ADHD score in the offspring was still evident, as was the association with risk of offspring having 6 or more ADHD symptoms. Adjusting for maternal dietary fiber intake did not change the effect estimates. High level of fiber intake has been suggested as a proxy for healthy diet [25]; however, our findings indicate that having an otherwise healthy diet did not completely eliminate the risks associated with SCB. Analyses using inverse probability weights to take into account loss to follow-up did not alter the findings, indicating that our estimates were not severely biased by this problem. This is in accordance with previous studies from Nordic birth cohorts, finding little impact on estimates when taking loss to follow-up into account [30, 34]. However, a recent study based on MoBa data found that some selection bias is likely for mental health associated exposures and outcomes [31]. Including maternal alcohol intake during pregnancy as an adjustment variable did not lead to major changes in the estimates, indicating that behaviours related to alcohol consumption during pregnancy did not bias our estimates.

A sibling analyses comparison based on discordant exposure (maternal intake of SCB) yielded somewhat higher coefficients, but less precise estimates than in the main analysis, with confidence intervals including zero for women drinking 1 or more servings SCB daily. Limitations of a sibling analysis comparison model include lower statistical power due to lower number of participants in these analyses, as the only sibling pairs that contribute to the estimate are pairs discordant on the exposure. Also, for MoBa, it required that the mother participated in MoBa with more than one pregnancy, and completed all the needed questionnaire data for this analysis (Q2 and Q8). This was reflected in the analysis by the wide confidence intervals. The possibility of non-shared confounding between siblings, where the sibling pairs discordant on the exposure also differ on other confounding variables, might further bias the analysis [35]. However, despite of these limitations, the positive coefficient in the sibling analysis supported the overall findings of an association between maternal SCB intake during pregnancy and offspring ADHD symptoms.

Our main aim was to study the potential effect of maternal intake of SCBs during pregnancy on offspring ADHD symptoms. The MoBa FFQ assessed habitual diet during the first half of pregnancy [21]. However, it has been reported that overall dietary habits appear to be relatively stable before, during and after pregnancy [36, 37]. A previous study from a smaller American pre-birth cohort (1235 mother–child pairs) reported similar associations for offspring cognitive outcomes for first and second trimester FFQs on maternal diet [16]. However, brain development continues throughout pregnancy and through adolescence and this development is vulnerable to later experiences. Animal studies feeding sucrose solutions to pregnant mice and young rats have indicated cognitive deficits and changes in the dopaminergic system, suggesting that sugar-sweetened beverages might affect the brain development at several stages.[15, 38]. While our main aim was to study the potential total effect of maternal intake of SCB during pregnancy on ADHD symptoms in the offspring, we also included an analysis with additional adjustment for offspring intake of sweetened beverages at 3 years of age. Offspring intake at 3 years cannot affect the exposure (maternal intake during pregnancy), but it can be regarded as a proxy for household routines and lifestyle associated with both maternal SCB intake and the risk of offspring ADHD and in this way represent a potential confounder. When adjusting for offspring intake of sweetened beverages, the associations were attenuated with no significant associations between maternal intake of SCB servings daily and offspring ADHD symptoms. If we view this adjustment as a way of taking into account household lifestyle, this attenuation suggests that there is no evident independent association between maternal intake of SCBs during pregnancy and offspring ADHD symptoms. However, this adjustment might also introduce bias as it might be on the causal path between our exposure and outcome [39].

Our results did attenuate somewhat when adjusting for maternal pre-pregnancy BMI and other maternal characteristics; however, the intake of 1 or more servings of SCB daily was still associated with ADHD symptoms. Intake of sugar-sweetened SCB contribute to the total energy intake. We adjusted for total energy intake to account for an overall higher energy intake among women with high SCB intake, which could possibly affect the outcome. The estimates were more profoundly attenuated when adjusting for total energy intake, indicating that for women with a similar total energy intake, high intake of SCB did possibly not contribute as much to further increase the risk of offspring ADHD symptoms compared to women with lower energy intake. However, while adjusting for energy intake estimated from FFQs could possibly infer some control for measurement error, the effects of this adjustment could be unpredictable [40]. When adjusting for energy coming from other sources than SCBs, nectars and fruit syrup, the coefficients and RRs were less attenuated. Adjusting for energy coming from other sources than SCBs, nectars and fruit syrup, reflects the association between the amount of SCB and offspring ADHD symptoms with SCBs as an addition to the diet, while adjusting for total energy reflects the association between SCBs and offspring ADHD symptoms emphasising the relative contribution of SCBs to the diet. It has been shown that liquid sugars do not reduce the consumption of other food [28, 29], and the model adjusting for energy contributed by other sources than SCBs, nectars and fruit syrup takes this into account.

Intake of SCB is a common dietary exposure, also in pregnancy, which makes it relevant for investigating potential effects on offspring health. Intake of sugar-sweetened beverages in the Norwegian population was historically high before and during the period of recruitment to MoBa, reaching a peak in 1997. After the millennium, the intake of artificially SCB has gradually increased, and in 2018, sales of artificially SCB made up 52% of the total SCB sales in Norway [14]. The observed association in the main analysis is a combination of the contribution from sugar-sweetened and artificially sweetened SCB. Stratifying the exposure into these two components yielded wide confidence intervals for both exposures with different attenuation of energy adjustment. For women with a daily intake of 1 or more servings daily our findings did, however, indicate that when adjusting for total energy intake, there was no additional risk for offspring ADHD symptoms related to sugar-SCB, while the increased risk remained for when adjusting for energy from other sources than SCBs, nectars and syrup. For artificially sweetened SCB the coefficients were around 0.3 but with confidence intervals including zero. However, with wide confidence intervals and imprecise estimates, more research on this topic is needed to draw conclusions.

Strengths of the study include the large sample size with detailed data both for the exposure and the outcome variables, the prospective design, minimizing recall bias and possible reverse causation, and the possibilities of additional sibling comparison analyses. The registration of ADHD symptoms in MoBa provides a continuous measured outcome. Previously, a high agreement between maternally reported offspring ADHD scores and registered offspring ADHD diagnoses from the Norwegian Patient Registry has been published [41]. The maternal reporting of symptoms avoids the potential bias of local/regional differences when diagnosing ADHD [42].

One of the limitations of the study is the inherent uncertainty linked to measures of dietary intake. There is no dietary assessment method without errors and measuring the true habitual intake is not feasible [22]. In MoBa, the FFQ was chosen as the preferred method to collect dietary data, rather than repeated 24-h recalls or using a food diary, based on considerations of social settings for the participants, the workload imposed on respondents, possibilities of validation and the economic burden on MoBa [21]. Still, the FFQ in MoBa has been found to be a valid tool for discriminating between high and low intakes of energy, nutrients and foods [22]. Another limitation in the study is the loss to follow-up from the baseline in the MoBa data. This could potentially bias the associations observed [43]. In an attempt to account for such bias, we created weights based on factors related to participation and loss to follow-up in MoBa. Our inverse probability weighting analysis did not indicate biased estimates by loss to follow-up. However, a recent study from MoBa exploring bias due to loss to follow-up, concluded that inverse probability weighting did not completely account for the selection bias in their exposure–outcome analyses. On the other hand, the authors also question if loss to follow-up might contribute to less confounding due to a more homogenous study population [44]. The sibling comparison analysis using data from women participating in MoBa with several pregnancies enabled us to investigate the potential influence of unmeasured, persistent familial confounding.

Maternal risk alleles for ADHD could be a potential confounder for the association between maternal diet and offspring ADHD. If the association between exposure and outcome seen in this study was confounded by genetics factors, associations could be evident without exposures being causal. The sibling comparison design would control for such genetic confounding. Further, we were able to exclude women reporting ADHD symptoms or having been diagnosed with ADHD to remove some of the possible genetic contribution to the associations. When including mothers with ADHD or ADHD symptoms, the estimates were somewhat stronger than the estimate from the main analyses adjusted for total energy, which might indicate some familiar confounding in the estimates or an interaction effect.

Women who consented to participate in the MoBa Cohort are older, more often nulliparous, higher educated and smoke less than Norwegian pregnant women in general [45]. In spite of this self-selection into MoBa, a previous study by Oerbeck et al. found that although affecting proportions of ADHD, psychosocial adversity and child global functioning, differences from the general population were small and association estimates with ADHD could be reasonably generalised to the total child population [46]. There was further some loss to follow-up from the baseline questionnaire until that at child’s age 8 years (Q8), and women who remained in the study were higher educated and were more often nulliparous than those completing Q1. If having a child with ADHD symptoms reduces the likelihood of completing questionnaires, women whose offspring had less ADHD symptoms might be better represented at Q8. However, our inverse probability weighting analysis did not indicate a biased estimate due to loss to follow-up, with results very close to the overall results.

Finally, MoBa is an observational study and conclusions about causality cannot be drawn with certainty, and residual confounding by for instance life-style factors or medical conditions not accounted for may be present. On the other hand, the longitudinal design with prospectively collected data, strengthens the results and their interpretation.

### Comparison with other studies

In humans, maternal diet and dietary components have previously been shown to be linked to neurodevelopmental outcomes in the offspring. In accordance with our finding, a systematic review and meta-analysis of 18 observational studies found that better maternal diet quality during pregnancy had a small positive association with child neurodevelopment [25]. A previous study using data from MoBa included in the review found that children whose mothers had unhealthy diets during pregnancy had higher levels of externalizing behaviour at 5 years of age. An unhealthy dietary pattern was characterized by high intake of processed meat products, refined cereals, sweet drinks and salty snacks [47]. Maternal intake of caffeine from soft drinks during pregnancy has been linked to offspring hyperactivity at 18 months of age [48]. However, a recent study found that the association between prenatal caffeine exposure and high activity at 18 months, 3 years, 5 years and 8 years was driven mainly by soft drink intake, not by the caffeine content [49]. Our results are comparable with these findings, indicating a small increased risk of ADHD symptoms at 8 year of age by high maternal consumption of SCB. We found no evident associations between other not-carbonated sweet drinks and offspring ADHD, and our results might reflect that women who consume carbonated sweet drinks report ADHD symptoms differently than women who do not consume carbonated drinks. Our findings are also compatible with a recent study from the MoBa cohort that found higher overall maternal diet quality to be associated with slightly lower ADHD symptom scores and risk of ADHD diagnoses in offspring aged 8 years [13]. The maternal diet quality was assessed using a composite index where added sugar was one of the components. Since this latter study is based on the same cohort as our, findings concerning the relation between maternal diet in pregnancy and offspring ADHD symptoms should be replicated in other populations before firm conclusions can be made.

In conclusion, in this large population-based cohort study, we found that maternal intake of SCB in pregnancy was associated with an increase in ADHD symptoms among offspring at 8 years of age. Attempting to control for unmeasured familiar factors in sibling analyses strengthened the evidence for an association between daily SCB intake and ADHD symptoms, but with wide confidence intervals. The magnitudes of associations in this study are weak, suggesting SCB only plays a minor role in the aetiology of ADHD. However, further research into causal agents is warranted, as SCBs are common exposures, and even a little reduction of risk may still be of importance for children’s ADHD symptoms at the population level.

## Supplementary Information

Below is the link to the electronic supplementary material.

Supplementary file1 (DOCX 92 KB)

## Data Availability

The consent given by the participants does not open for storage of data on an individual level in repositories or journals. Researchers who want access to data sets for replication should submit an application to datatilgang@fhi.no. Access to data sets requires approval from The Regional Committee for Medical and Health Research Ethics in Norway and an agreement with MoBa.

## Code availability

Available upon request.
