# Supplementary material for: Association of sweetened carbonated beverage consumption during pregnancy and ADHD symptoms in the offspring: a study from the Norwegian Mother, Father and Child Cohort Study (MoBa)
Source: Eur J Nutr. 2022 Jan 23;61(4):2153–66. doi: 10.1007/s00394-022-02798-y (PMC9106638; doi:10.1007/s00394-022-02798-y)
Supplement: Supplementary file 1 — Supplementary file1 (DOCX 92 KB) [file 394_2022_2798_MOESM1_ESM.docx]

**Supplemental material**

### Association of sweetened carbonated beverage consumption during pregnancy and ADHD symptoms in the offspring. A study from the Norwegian, Mother, Father and Child Cohort Study (MoBa).

### Liv G Kvalvik (ORCID: 0000-0001-6520-9057), Kari Klungsøyr (ORCID: 0000-0003-2482-1690), Jannicke Igland (ORCID: 0000-0002-2289-0978), Ida Henriette Caspersen (ORCID: 0000-0003-2591-8435), Anne Lise Brantsæter(ORCID: 0000-0001-6315-7134), Berit Skretting Solberg (ORCID: 0000-0002-0168-1675), Catharina Hartman (ORCID: 0000-0002-8094-8859), Lizanne Johanna Stephanie Schweren (ORCID: ORCID: 0000-0001-6018-5316), Henrik Larsson (ORCID: 0000-0002-6851-3297) , Lin Li (ORCID:0000-0002-7946-4574) , Ingeborg Forthun (ORCHID: 0000-0001-8097-6959), Stefan Johansson (ORCID:0000-0002-2298-7008), Alejandro Arias Vasquez (ORCID: 0000-0002-4786-0169), Jan Haavik. (ORCID: 0000-0001-7865-2808).

### **Supplementary Table 1.** Poisson models for the association between maternal intake of sweetened carbonated beverages (SCB) during pregnancy and offspring ADHD symptoms at 8 years of age with adjustment for maternal depression and anxiety score around gestational week 30 (in Questionnaire 3 (Q3)).

|  |  |  |  |  | Relative risk (RR) of offspring having 6 or more ADHD symptoms at 8 years of age (95% CI) § | | | | | |
| --- | --- | --- | --- | --- | --- | --- | --- | --- | --- | --- |
| Maternal intake of SCB during pregnancy (daily intake) | Study population total (mother-child pairs) | N (%) offspring with 6 or more ADHD symptoms | N Total mother-child pairs where mothers also answered Q3 | N (%) offspring with 6 or more ADHD symptoms where mothers also answered Q3 | Unadjusted RR (95% CI) | Unadjusted RR (95% CI) when restricted to participation in Q3 | Adjusted RR (95% CI) ^a^ | Adjusted RR (95% CI) ^b^ | Male offspring  Adjusted RR (95% CI) ^c^ | Female offspring  Adjusted RR (95% CI) ^c^ |
| <1 serving daily | 34 469 | 1 165 (3.4) | 33 959 | 1 143 (3.4) | 1 (reference) | 1 (reference) | 1 (ref) | 1 (ref) | 1 (ref) | 1 (ref) |
| 1 serving or more daily | 5 277 | 236 (4.5) | 5 190 | 234 (4.5) | 1.32 (1.15, 1.52) | 1.34 (1.17, 1.54) | 1.16 (1.004, 1.34) | 1.20 (1.04, 1.38) | 1. 23 (1.04, 1.46) | 1.03 (0.78, 1.35) |

§ A modified Poisson regression model with a robust error variance was used to estimate the relative risks and 95% confidence intervals

^a^ Adjusted for daily intake of other sweet beverages in Q2 such as nectars and fruit syrup, and total fiber intake, for maternal education, age, parity, prepregnancy BMI, for birth year, birth season, energy intake and maternal depression/anxiety.

b Adjusted as above but with energy from other sources than SCBs, nectars and fruit syrup instead of total energy adjustment.

c Stratified on offspring gender, same adjustment as in model marked with ^a^.

**Supplemental Table 2.** Regression coefficients and 95% CIs from linear mixed models for the association between maternal intake of sweetened carbonated beverages (SCB) in three categories and a standardized ADHD offspring symptoms score. Total n=39 760 §

|  | Unadjusted coefficient (95% CI) ^a^ | Adjusted coefficient (95% CI) without total energy intake adjustment ^b^ | Adjusted coefficient (95% CI) with total energy intake adjustment ^c^ | Adjusted coefficient (95% CI) with adjustment for energy intake other than from SCBs, nectars and fruit syrup ^d^ |
| --- | --- | --- | --- | --- |
| A model with three categories of SCB intake |  |  |  |  |
| <1 serving daily | 0 (ref) | 0 (ref) | 0 (ref) | 0 (ref) |
| 1 serving daily | 0,27 (-0.20, 0.74) | 0.15 (-0.32, 0.63) | -0.01 (-0.48, 0.46) | 0.04 (-0.43, 0.51) |
| 2 or more servings daily | 1.18 (0.81, 1.55) | 0.78 (0.40, 1.16) | 0.49 (0.10, 0.87) | 0.68 (0.30, 1.06) |

a Unadjusted regression coefficient from linear mixed model with the ADHD scores standardized to a mean of 50 and a standard deviation of 10 as outcome, intake of SCB as fixed effect and random intercept for mother (n=39 760).

b Regression coefficient from linear mixed model adjusted for daily intake of other sweet beverages such as nectars and fruit syrup, total fiber intake, maternal education, age, parity, prepregnancy BMI, maternal depression and anxiety at gestational week 30, birth year and birth season (n= 37 353 complete cases).

c As above but additional adjustment for total energy intake (kcal).

d As in model b, but with additional adjustment for total energy intake other than contributed from SCBs, nectars and fruit syrup.

**Supplementary Table 3**. Mother-child units (singleton pregnancies) participating in MoBa in baseline questionnaire Q1, in Q2, and followed up from Q2 to Q8.

|  | Whole cohort (answered baseline questionnaire, Q1) | Cohort who answered food frequency questionnaire (Q2) | Cohort who answered Q2 and questionnaire when offspring was 8 years (Q8) | Study population who answered Q2 and Q8 |
| --- | --- | --- | --- | --- |
| Mother- child units | 100 212 | 85 974 | 41 187 | 39 870 |
|  | N (%) | N(%) | N (%) | N (%) |
| Maternal age at delivery (years) |  |  |  |  |
| <20 | 974 (1.0) | 762 (0.9) | 193 (0.5) | 176 (0.4) |
| 20-24 | 10 128 (10.1) | 8 544 (9.9) | 3 111 (7.6) | 2 925 (7.3) |
| 25-29 | 32 929 (32.9) | 28 199 (32.8) | 13 187 (32.0) | 12 801 (32.1) |
| 30-34 | 38 440 (38.4) | 33 209 (38.6) | 16 801 (40.8) | 16 344 (41.0) |
| 35 years and older | 17 400 (17.4) | 15 092 (17.6) | 7 895 (19.2) | 7 624 (19.1) |
| Missing maternal age | 341 (0.3) | 168 (0.2) | - | - |
| Maternal parity |  |  |  |  |
| 0 | 44 734 (44.6) | 39 200 (45.6) | 18 852 (45.8) | 18 273 (45.8) |
| 1 | 35 549 (35.5) | 30 303 (35.3) | 14 530 (35.3) | 14 064 (35.3) |
| 2+ | 19 588 (19.6) | 16 303 (19.0) | 7 805 (19.0) | 5 533 (18.9) |
| Missing parity | 341 (0.3) | 168 (0.2) | - | - |
| Maternal education from Q1 |  |  |  |  |
| Less than high school | 3 188 (3.2) | 2 464 (2.9) | 669 (1.6) | 599 (1.5) |
| High school | 34 003 (33.9) | 28 109 (32.7) | 11 453 (27.8) | 10 903 (27.4) |
| 4 years or more of college/university | 60 888 (60.8) | 52 995 (61.6) | 28 183 (68.4) | 27 522 (69.0) |
| Missing | 2 133 (2.1) | 2 406 (2.8) | 882 (2.1) | 840 (2.1) |
| Intake of SCB during mid-pregnancy |  |  |  |  |
| <1 serving daily | 87 093 (86.9) | 72 737 (84.6) | 35 670 (86.6) | 34 571 (86.7) |
| 1 serving daily | 4 035 (4.0) | 4 063 (4.7) | 1 814 (4.4) | 1 765 (4.4) |
| 2-3 servings daily | 5 215 (5.2) | 5 265 (6.1) | 2 191 (5.3) | 2 108 (5.2) |
| 4 or more servings daily | 3 869 (3.9) | 3 909 (4.5) | 1 512 (3.7) | 1 426 (3.6) |

**Supplementary Figure 1.** A directed acyclic graph (DAG) outlining the association between maternal intake of artificially and/or sugar-sweetened carbonated beverages during pregnancy and child ADHD symptoms at 8 years of age. Child’s intake of artificially and/or sugar-sweetened beverages at 3 years of age is an intermediate variable between the exposure and the outcome, but could also be a proxy for household behavior (thus representing a potential confounder).


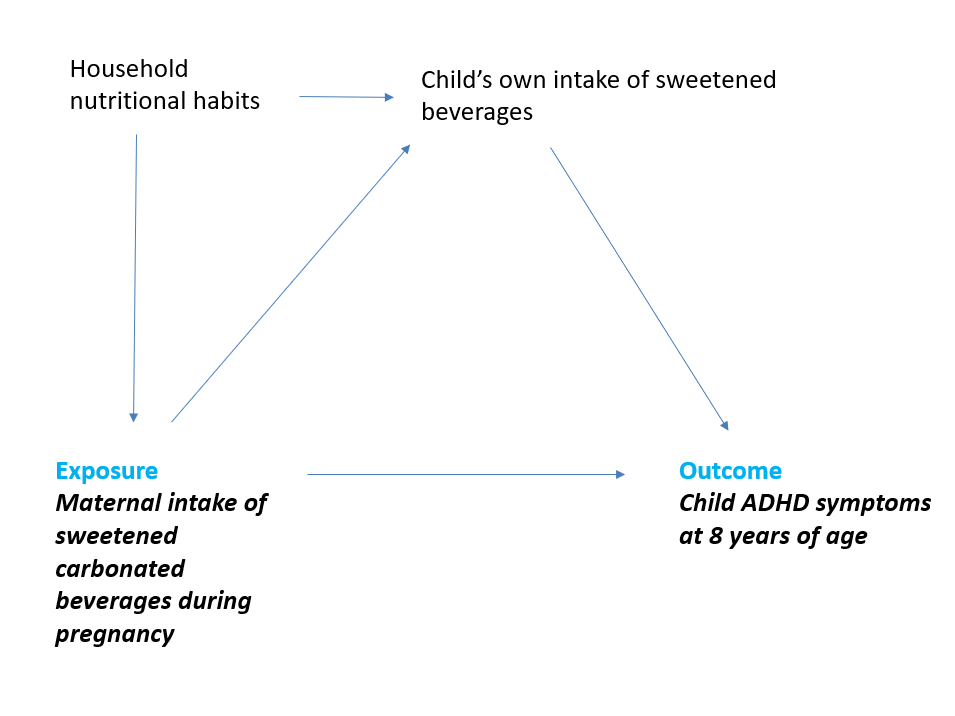


**Supplementary Table 4.** Regression coefficients and 95% CIs from linear mixed models for the association between maternal intake of sweetened carbonated beverages (SCB), nectars and fruit syrup and a standardized ADHD offspring symptoms score. Total n=39 760§

|  | Adjusted coefficient (95% CI) without total energy intake adjustment ^a^ | Adjusted coefficient (95% CI) with total energy intake adjustment ^b^ | Adjusted coefficient (95% CI) with adjustment for energy intake other than from SCBs, nectars and fruit syrup ^c^ |
| --- | --- | --- | --- |
| A model with SCBs as exposure and nectars/fruit syrup as separate exposures in the same model |  |  |  |
| <1 serving daily of SCB | 0 (ref) | 0 (ref) | 0 (ref) |
| 1 or more serving daily of SCBs | 0.56 (0.25, 0.87) | 0.31 (0.001, 0.62) | 0.46 (0.15, 0.77) |
| <1 serving of nectars or fruit syrup daily | 0 (ref) | 0 (ref) | 0 (ref) |
| 1 or more serving of nectars or fruit syrup daily | 0.05 (-0.17, 0.27) | -0.17 (-0.39, 0.05) | 0.02 (-0.20, 0.23) |
|  |  |  |  |
| A model with SCBS, nectars and fruit syrup as a combined exposure |  |  |  |
| <1 serving of SCBs, nectars or fruit syrup daily | 0 (ref) | 0 (ref) | 0 (ref) |
| 1 or more serving of SCBs, nectars or fruit syrup daily | 0.22 (0.02, 0.42) | -0.03 (-0.24, 0.17) | 0.16 (-0.05, 0.36) |

§ For mother-offspring units to join the analyses 9 or more out of 18 questions on ADHD symptoms were required to be answered. For n=110 (0.3%), the mothers had answered 8 or less questions and these mother-offspring units were excluded from the analyses.

a Regression coefficient from linear mixed model adjusted for daily intake of other sweet beverages such as nectars and fruit syrup (included when nectars and fruit syrup was a covariate in the model, and not included as adjustment when these beverages were part of the exposure), total fiber intake, maternal education, age, parity, prepregnancy BMI, maternal depression and anxiety at gestational week 30, birth year and birth season.

b As above but additional adjustment for total energy intake (kcal).

c As above but additional adjustment for total energy intake other than contributed from SCBs, nectars and fruit syrup
